# Factors associated with muscle strength in 10–16-year-old trained male children and adolescents

**DOI:** 10.1186/s13102-025-01272-6

**Published:** 2025-08-15

**Authors:** Daniel Jansson, Magnus Domellöf, Helena Andersson, Apostolos Theos, Elena Lundberg

**Affiliations:** 1 Department of Community Medicine & Rehabilitation, Section of Sports Medicine, Umeå University, Linnaeus väg 9, Umeå, 90187 Sweden; 2 Department of Clinical Sciences, Pediatrics, Umeå University, Umeå, Sweden

**Keywords:** Maturation, Leg press, Bench-press, Growth, Pediatrics

## Abstract

**Background:**

This study investigated the associations of muscular strength measures with anthropometry, chronological age, biological maturation, and training experience in trained prepubertal and pubertal males. Another aim was to investigate if handgrip strength can predict general or overall muscle strength in the same population.

**Method:**

Forty-one (*n* = 41) trained male children and adolescents aged 10–16 participated in the study. The 10-repetition maximum (RM) leg press and bench press were used to assess upper- and lower-body muscular strength, handgrip strength was used as an overall strength assessment, and a countermovement jump with arm swing (CMJa) was used to estimate extensor muscle power of the lower extremity. The maturity status was determined using the Tanner scale. Anthropometric factors included height, body mass, two skinfolds, limb length, and lean leg volume. Multivariable linear regressions were performed on absolute strength values to explore predictors of muscular strength and power.

**Results:**

Body mass explained 81% of the variance in leg press strength (*p* < 0.001), whereas bench press was associated with body mass and chronological age, explaining 83% of the variance (*p* < 0.001). The countermovement jump (CMJa) height was positively associated with lean leg volume, which explained 52% of the variance (*p* < 0.001). Chronological age and fat-free mass explained 87% of the variance in handgrip strength (*p* < 0.001). Biological maturity (Tanner) did not contribute to the final models. Handgrip strength was strongly associated with total muscle strength (*r* = 0.89–0.91, *p* < 0.001).

**Conclusion:**

The results indicate that anthropometrical factors, rather than biological maturity, are associated with muscular strength in trained male children and adolescents. Our findings suggest that handgrip strength may be a quick and effective screening tool for assessing total muscle strength in youth.

**Supplementary Information:**

The online version contains supplementary material available at 10.1186/s13102-025-01272-6.

## Introduction

Understanding maturity and growth-related changes in physical performance is important to ensure that young children receive high-quality coaching and training [1]. Maturation timing can influence both performance and health, as late-maturing children may experience fewer sport-specific development opportunities, such as selection to competitive teams, access to advanced training, or early talent identification, compared with their early-maturing peers [2]. One key aspect of physical development is muscle strength, which plays a crucial role in athletic performance and overall health. Muscle strength testing is frequently used to monitor strength development, assess muscular fitness, and evaluate recovery after injury throughout childhood [3]. Furthermore, recent reports have indicated a decline in children’s muscular fitness, with contemporary children being significantly weaker than previous generations [4, 5]. This decline is concerning, as low muscular strength is linked to elevated metabolic risk factors [6], whereas high levels of muscle strength enhance performance in fundamental movement patterns such as sprinting, change of direction, throwing, and vertical jumping [7–9]. Therefore, understanding the factors influencing muscle strength across various exercises and muscle groups is essential for researchers and practitioners to create a developmentally appropriate training environment for young athletes.

Factors associated with strength during childhood development often illustrate heightened muscular strength with chronological age and maturation [10–12]. Children of the same chronological age can vary significantly in their growth and development, which may be better reflected in the measures of biological maturity. Some studies have suggested that biological maturity is a more critical independent variable in the development of muscular strength than simple chronological age since it considers a child’s inter-individual development [13]. The relationship between maturation and strength development seems to increase linearly until two years after peak-height velocity and then levels off and may even reach a plateau [13]. However, although maturity and age are significantly associated with muscular strength, they seem less important when examined concurrently with anthropometrical factors (e.g., body mass and thigh volume) [13].

Anthropometrical characteristics of youth are typically positively associated with muscular strength levels [3]. Body mass and height are the most commonly used anthropometrical variables associated with muscular strength in children and adolescents [13–15]. They are easy to assess and show high correlations with muscular strength levels in children and adolescents [13, 14], and hence, they are often used by researchers and practitioners. Additionally, body composition variables such as fat-free mass (FFM) are another factor used to predict muscle strength, although less commonly used [3, 16]. FFM measures the lean components of the body (muscles, bones, organs, and connective tissue) and is, therefore, preferred because muscles are the primary contributors to strength. Previous studies have shown a relationship between muscle strength and FFM, although it seems to vary with age, potentially being more significant in younger than in older children [3].

A methodological challenge in pediatric exercise science research is accounting for differences in body size when comparing performance (e.g. strength) across children and adolescents. Normalization techniques, such as ratio scaling (e.g. dividing strength by body mass) or allometric scaling (e.g. strength/body mass^k^) aim to reduce the influence of body size and enable more meaningful comparisons [17]. Proper normalization allows researchers to determine whether greater strength is due to actual physiological capacity or simply differences in body mass or composition [17]. Despite the importance of scaling, few studies have applied or compared different scaling methods in relation to specific strength tests in pediatric populations.

Previous studies examining factors related to muscular strength in the pediatric population have primarily focused on field tests, such as handgrip strength [11], sit-ups [14, 18], standing broad jump [14] and vertical jump [16, 19] with limited studies assessing loaded resistance training exercises. Given that resistance training with external loads (e.g. machine-based exercises or free-weight exercises) in youth has shown promise for enhancing both health (e.g. bone health) and performance (e.g. strength, motor skills) [20], our study includes outcomes to assess lower- and upper-body muscular strength with loaded resistance training exercises (e.g., leg press and bench press). Understanding the factors underpinning muscle strength development in common strength tests and exercises may provide valuable insights for coaches, researchers, and trainees to better understand children’s inter-individual strengths and limitations, thereby enabling a fair interpretation of test and training scores. In addition, if a simple and easily administered handgrip strength test can reliably predict total muscular strength assessed through exercises like the leg press and bench press, it would serve as a practical screening tool. Such a test requires minimal equipment, limited time, and little prior experience from the test administrator.

This study is among the first to analyze how anthropometric, maturity, and body size variables relate to muscular strength in loaded resistance exercises among trained male youth athletes. It also explores whether strength outcomes are exercise-specific and whether handgrip strength can act as a reliable predictor of total muscular strength. These findings could help inform athletes testing, screening, and individualized programming in youth sports.

## Materials and methods

### Experimental approach to the problem

Cross-sectional data from a previous clinical trial [21] were used to investigate the influence of growth, maturation, and anthropometry on muscular strength and estimates of lower-limb power in trained male children. Univariable and multivariable regression models were used to examine the factors related to muscular strength and lower-limb muscular power. The participants attended two laboratory sessions at similar times during the day. The first session consisted of three parts: (I) an overview of the study, (II) warm-up and familiarization with the exercise protocols and equipment, and (III) anthropometric measurements. The second session was conducted at least two days later and included (I) body composition measurements and (II) muscular strength tests. All sessions started with a warm-up, including a 5-minute cycling on a cycle ergometer (60 W), followed by a 5-10-minute whole-body dynamic warm-up involving exercises such as squats, lunges, leg swings, and push-ups.

### Participants

A total of 41 healthy male children from local athletic clubs participated in this study. Details from the participant flow chart and recruitment were reported in another study [21]. The children, ranging in age from 10 to 16 years, participated in a minimum of 4–5 organized training sessions per week for the youngest and up to 6–8 sessions per week for the eldest. The training sessions lasted 60 to 90 min. Information on the participants’ weekly training frequency, session duration, sport types, and training history was collected using a structured questionnaire completed by both the child and a parent. The data were entered and managed in a spreadsheet format for further analysis. Participants had diverse training backgrounds, many participating in multiple sports, primarily engaging in team sports such as soccer, floorball, and volleyball for at least two years. This study exclusively focused on male children, utilizing pre-collected data as described elsewhere [21]. The participants were healthy and free from injuries that could hinder their full participation in the study. The study included youth athletes with limited experience in resistance training with free weights or machine-based exercises. The testing procedures were fully explained, and both participants and their legal guardians provided informed consent. The study was approved by the Swedish Ethical Review Board (Nr: 2020–03179),

### Data collection

#### Anthropometric and body composition measurement

Body mass (kg) was measured by using a digital scale (SECA, UK). Height (cm) was measured using a wall-mounted stadiometer (SECA, UK) to the nearest 0.1 cm. The skinfold thickness was assessed using a Harpenden skinfold caliper (British Indicators Ltd, St Albans, Herts.) at two sites (triceps and subscapular). Calculations were performed to estimate fat-free mass (FFM) using the skinfold thickness of the triceps and subscapular regions by applying the commonly used Slaughter et al. [22] youth-specific equation. Additionally, lean leg volume (LLV) was determined by measuring leg height and circumference using a previously validated anthropometric model by Jones and Pearson [23]. All anthropometrical variables were measured in duplicate, and the mean values were used for the analysis. The same test leader trained in anthropometry measured all anthropometrical assessments to minimize variability.

#### Biological maturation

Biological maturation was assessed by an experienced physician according to the Tanner (I-V) scale [24] and the Prader orchidometer [25]. Tanner I-II was considered prepubertal, and Tanner III-IV was classified as pubertal.

#### Muscular strength assessment

Four tests were used to assess muscle strength and power: 10 RM leg press, 10 RM bench press, handgrip strength, and countermovement jump (CMJa). Lower-body strength was evaluated using a horizontal leg press machine (Agaton Fitness AB, Boden, Sweden), while upper-body strength was assessed with a 10 RM barbell bench press (Eleiko, Halmstad, Sweden). The 10 RM protocol was selected instead of 1 RM or 3 RM testing due to safety considerations for young participants, as higher repetition maximum tests reduce the risk of excessive joint and muscle strain. A certified Strength and conditioning coach supervised all strength assessments.

The 10 RM was defined as the maximum resistance lifted for 10 repetitions with full range of motion and proper technique. For the leg press, good form was defined as maintaining controlled movement without excessive lumbar flexion and keeping both feet flat on the platform throughout the lift. Range of motion was ensured by setting a knee angle of approximately 90° at the lowest position, which researchers visually monitored during each trial. Bench press: Good form required participants to maintain five points of contact (head, upper back, and glutes on the bench, feet flat on the floor, and hands gripping the bar at a consistent width). Full range of motion was defined as lowering the barbell to lightly touch the chest and pressing it back to full elbow extension without bouncing the bar off the chest or lifting the hips off the bench. Range of motion and technique were visually monitored by researchers to ensure consistency.

Prior to testing, participants followed a standardized warm-up protocol consisting of: (1) 8–12 repetitions with light loads, (2) 10 repetitions with a relatively light load, (3) 10 repetitions with a moderately heavy load, and (4) progressive 10 RM attempts until failure. Load increments of at least 0.5 kg were applied for successful attempts. In most cases, the 10 RM was determined within four to six attempts. To aid in selecting the appropriate load, participants rated their perceived exertion after each set using a 10-point scale adapted for children [26]. The children attended a familiarization session prior to the actual strength testing, during which they were introduced to the scale. Total muscle strength was estimated by summing the 10 RM values from the leg press and bench press. This approach was used to provide a composite measure of total-body dynamic strength, reflecting both upper- and lower-body capabilities. Previous studies have used similar summation methods to assess overall strength in youth populations [27].

Handgrip strength was assessed according to a previously validated and reliable protocol (ICC > 0.80) [27]. The test was conducted with the participant seated, with the shoulder adducted, and flexed at 70 degrees [27, 28]. Handgrip strength was measured in both the dominant and non-dominant arms using a digital handgrip dynamometer (Jamar Plus+, Patterson Medical, Warrenville, IL, USA). Standardized encouragement was provided to the participants during the test, and each participant performed three trials for each arm, with a 2-minute rest period between trials. The coefficients of variation (CV) was 4.3% and 5.9% for the dominant and non-dominant hand, respectively. ICC was 0.99 (95% CI, 0.98–0.99) and 0.98 (95% CI, 0.98–0.99) for the dominant and non-dominant hands, respectively. The highest value obtained for the dominant arm was used in the regression analysis.

#### Vertical jump assessment

Following a previously validated procedure, lower-limb muscular power was estimated using jump height from a standardized countermovement jump with arm swing (CMJa) [29]. The OptoJump system (Optojump, Microgate, Bolzano, Italy), previously validated in a study [30], was used to measure jump height. Participants conducted a minimum of three to a maximum of five jumps. The coefficient of variation (CV) between the jumps was 2.8%. The ICC was 0.98 (95% CI; 0.96–0.99). The highest recorded jump height was selected for subsequent analysis.

### Statistical analysis

Descriptive statistics are presented as the mean ± SD. Normality was tested using the Shaprio-Wilks test and visual inspection of histograms. Outcome variables that were not normally distributed were log-transformed before statistical analysis. Comparisons between prepubertal and pubertal males for each variable were performed using an independent Student T-test. Moreover, to allow for comparison between maturity groups, the strength variables were ratio and allometrically scaled to body size using methods reported elsewhere [31]. The reliability of the vertical jump test (CMJa) and handgrip strength test was assessed by calculating intraclass correlation coefficients (ICCs) and their 95% confidence intervals. According to previous guidelines, an ICC of 0.90 was interpreted as high reliability for physiological field tests [32]. Pearson’s correlation coefficient was used to assess the correlation between total muscle strength and handgrip strength. Univariable and multivariable regression models were used to examine possible associations between the dependent variable (muscle strength measures) and the independent variables (anthropometry, maturation, chronological age, and training experience). The correlation coefficients were interpreted according to Cohen [33]; a correlation coefficient of *r* < 0.3 is considered weak, 0.3 < *r* < 0.5 moderate, and *r* > 0.5 strong. The model was refined using the backward elimination method, where all possible predictors were included in the initial model and subsequently removed one non-significant variable at a time until all significant variables remained. The final model was further examined for collinearity between predictors using the variance inflation factors (VIF). If high collinearity was found between variables (VIF > 5), the variable explaining the greatest proportion of variance was retained [34]. The significance level was set at α = 0.05. Additionally, we reported the effect size according to partial eta squared (η^2^), which was considered small (~ 0.01), moderate (~ 0.06), or large (≥ 0.14) [33]. All statistical analysis were performed using the SPSS statistical package (SPSS, v. 24, Chicago, IL).

## Results

### Participant characteristics

The descriptive data of the participants are summarized in Table 1. Large variations were noted in body mass (range: 25.8–88.5 kg), and height (range: 129.3–201.6 cm). Based on Tanner stages, participants’ pubertal stage ranged from 1 to 5 (Tanner stages; I: *n* = 17; II, *n* = 4; III = 5; IV, *n* = 10 and V: *n* = 5); thus, the sample spanned from prepubertal (Tanner I-II, *n* = 21) to pubertal children (Tanner III-IV, *n* = 20). Splitting the data based on maturation (prepubertal vs. pubertal) showed that all anthropometrical variables were higher in the pubertal group (all, *p* < 0.05). The pubertal group was heavier, taller, and had a greater fat-free mass than their less mature counterparts (*p* < 0.05). There was also a high variation in the participants’ training experience, ranging from 2 to 12 years of participating in organized sports.

**Table 1:** Descriptive statistics for participants’ anthropometric and maturity characteristics assessments (*n* = 41)

	Prepubertal (*N* = 21)	Pubertal (*N* = 20)	All (*N* = 41)
Chronological age (years)	11.4 ± 1.1	15.7 ± 0.6*	13.5 ± 2.4
Body mass (kg)	42.2 ± 7.9	66.4 ± 8.6*	53.9 ± 14.7
Height (cm)	150.6 ± 8.8	179.2 ± 7.0*	164.6 ± 16.4
Fat-free mass (kg)	33.3 ± 5.3	57.8 ± 6.8*	45.2 ± 13.7
Leg length (cm)	69.8 ± 6.1	86.0 ± 4.3*	77.5 ± 9.7
Body fat (%)	12.6 ± 3.0	17.7 ± 3.3*	17.2 ± 7.3
Lean Leg Volume (L)	3.6 ± 0.9	7.3 ± 0.7*	5.4 ± 2.1
Training experience (years)	5.3 ± 1.3	8.1 ± 0.9*	6.6 ± 2.4

Values are presented as mean ± SD

* Significantly different between prepubertal and pubertal males; *p* < 0.05

### Influence of maturation on muscle strength

The influence of maturation on muscle strength are summarized in Table 2 and presented in absolute values, ratio-scaled (body mass) values and allometrically scaled to body mass for each test.

#### Leg press

Pubertal children demonstrated significantly higher absolute leg press strength than prepubertal children (*p* < 0.001), with a large effect size (η² = 0.612) and strong positive correlation with body mass (*r* = 0.90, *p* < 0.001). Normalizing strength with ratio-scaling (kg/BM) was also significantly greater in the pubertal group than in the prepubertal group (*p* < 0.001), with a moderate effect size (η² = 0.312) and a correlation with body mass (*r* = 0.58, *p* < 0.001). After allometric scaling to BM^1.36^ no significant differences were found between the groups (*p* = 0.137), with a small effect size (η² = 0.056) and weak correlation with body mass (*r* = 0.18, *p* = 0.253).

#### Bench-press

In absolute values, pubertal children showed significantly greater bench press strength than prepubertal children (*p* < 0.001), with a large effect size (η² = 0.697) and a strong correlation with body mass (*r* = 0.88, *p* < 0.001). Ratio-scaled values (kg/BM) were also significantly higher in pubertal children than in prepubertal children (*p* < 0.001; η² = 0.532) and were strongly correlated with body mass (*r* = 0.67, *p* < 0.001). Allometrically scaled values to BM^1.08^ were still significantly higher in pubertal children than in prepubertal children (η² = 0.488; *p* < 0.001) with a strong correlation (*r* = 0.61, *p* < 0.001). However, using FFM^1.45^ rather than BM as the scaling variable resulted in no significant differences between groups (prepubertal 0.10 ± 0.01 vs. pubertal 0.11 ± 0.02, n^2^ = 0.011; *p* = 0.52) with small correlation to FFM (*r* = 0.04, *p* = 0.79).

#### Handgrip strength

Pubertal children showed significantly greater handgrip strength in absolute values than prepubertal children (*p* < 0.001), with a large effect size (η² = 0.729) and a strong correlation with body mass (*r* = 0.90, *p* < 0.001). Ratio-scaled handgrip strength (kg/BM) was significantly higher in pubertal children than in prepubertal children (η² = 0.466; *p* < 0.001) and moderately correlated (*r* = 0.598, *p* < 0.001). Allometric scaling to BM^0.91^ maintained significant differences (η² = 0.572; *p* < 0.001) between pubertal and prepubertal children and correlated with body mass (*r* = 0.632, *p* < 0.001). However, using FFM^1.38^ rather than BM as the scaling variable resulted in no significant differences between groups (prepubertal 0.16 ± 0.03 vs. pubertal 0.17 ± 0.03, η² = 0.005; *p* = 0.64) with small correlation to FFM (*r* = 0.017, *p* = 0.92).

#### CMJa

absolute jump height was significantly higher in pubertal children than in prepubertal children (*p* < 0.001), with a moderate effect size (η² = 0.391) and moderate correlation with body mass (*r* = 0.609, *p* < 0.001). Ratio-scaled values (cm/BM) showed no significant difference between the groups (*p* = 0.565), with a negligible effect size (η² = 0.009) and a negative correlation with body mass (*r* = -0.308, *p* = 0.05). Allometric scaling to BM^0.31^ revealed a significant difference between pubertal and prepubertal children (*p* = 0.001), with a small effect size (η² = 0.239) and moderate correlation (*r* = 0.416, *p* = 0.007). However, using LLV^0.62^ rather than BM as the scaling variable resulted in no significant differences between groups (prepubertal 12.6 ± 1.89 vs. pubertal 12.3 ± 3.47, η² = 0.003; *p* = 0.87) with a small correlation to lean leg volume (*r* = 0.02, *p* = 0.87).

**Table 2:** Leg press, bench-press, handgrip strength, dominant arm, and countermovement jump (CMJa) height in trained prepubertal and pubertal children. Values are presented in absolute values, ratio-scaled, and allometrically scaled to body mass

	Prepubertal (*N* = 21)	Pubertal (*N* = 20)	*p*-value	Effect size (*n*^2^)	Strength variable vs. Body size scaling variable (*r*)
*Leg press*,* 10 RM*					
Absolute value (kg)	50.0 ± 16.2	101.8 ± 25.3	< 0.001	0.612	*r* = 0.90, *p* < 0.001
Ratio-scaled (kg/BM)	1.17 ± 0.24	1.52 ± 0.29	< 0.001	0.312	*r* = 0.58, *p* < 0.001
Allometric scaling (kg/BM^1.36^)	0.30 ± 0.1	0.33 ± 0.1	0.137	0.056	*r* = 0.18, *p* = 0.253
*Bench-press*,* 10 RM*					
Absolute value (kg)	17.4 ± 4.2	40.5 ± 10.3	< 0.001	0.697	*r* = 0.88, *p* < 0.001
Ratio-scaled (kg/BM)	0.41 ± 0.06	0.61 ± 0.12	< 0.001	0.532	*r* = 0.67, *p* < 0.001
Allometric scaling (kg/BM^1.08^)	0.30 ± 0.05	0.43 ± 0.08	< 0.001	0.488	*r* = 0.61, *p* < 0.001
Allometric scaling (kg/FFM^1.45^)	0.10 ± 0.01	0.11 ± 0.02	0.52	0.011	*r* = 0.04, *p* = 0.79
*Handgrip*,* dominant arm*					
Absolute value (kg)	21.6 ± 6.4	47.2 ± 9.4	< 0.001	0.729	*r* = 0.90, *p* < 0.001
Ratio-scaled (kg/BM)	0.51 ± 0.11	0.71 ± 0.10	< 0.001	0.466	*r* = 0.598, *p* < 0.001
Allometric scaling (kg/BM^0.91^)	0.71 ± 0.15	1.03 ± 0.15	< 0.001	0.572	*r* = 0.632, *p* < 0.001
Allometric scaling (kg/FFM^1.38^)	0.16 ± 0.03	0.17 ± 0.03	0.64	0.005	*r* = 0.017, *p* = 0.92
*Countermovement jump (CMJa)*					
Absolute value (cm)	27.3 ± 4.9	41.7 ± 12.3	< 0.001	0.391	*r* = 0.609, *p* < 0.001
Ratio-scaled (cm/BM)	0.66 ± 0.16	0.63 ± 0.19	0.565	0.009	*r* = -0.308, *p* = 0.05
Allometric scaling (cm/BM^0.31^)	8.6 ± 1.5	11.4 ± 3.3	0.001	0.239	*r* = 0.416, *p* = 0.007
Allometric scaling (LLV^0.62^)	12.6 ± 1.89	12.3 ± 3.47	0.87	0.003	*r* = 0.02, *p* = 0.87

Values are presented as mean ± SD

### Predictors of muscle strength

Table **S1** (Supplementary Material) shows the results of univariable linear regression analyses. All ten variables (Table **S1**) showed a positive relationship with all the dependent variables (leg press, bench press, CMJa, and handgrip strength). The associations ranged from moderate to strong effects (β = 0.28 to 0.91) for leg press and bench press strength. The single strongest predictor for 10 RM leg press was body mass (β = 0.90, *p* < 0.001), while FFM was the strongest predictor for 10 RM bench press (β = 0.91, *p* < 0.001). The linear regressions ranged from moderate to large effects (β, 0.48 to 0.93, *p* < 0.01) for handgrip strength, with the highest predictor being FFM (β = 0.93, *p* < 0.001). Associations between lower-limb muscular power (CMJ) and the independent variables ranged from moderate to large effects (β = 0.30 to 0.69), with the highest predictive power observed between CMJa and LLV (β = 0.72, *p* < 0.001).

The multivariable linear regression analyses for each dependent variable are presented in Table 3. None of the independent variables in the final model showed collinearity (VIF < 5). In the final model of leg press strength, only body mass was retained, which explained 81% of the variation in performance (*p* < 0.001). Body mass, and chronological age together explained 82% of the variation in bench press strength (*p* < 0.001). In the final CMJa model, only LLV was included and explained 51% of the variation (*p* < 0.001). The handgrip strength model included chronological age and fat-free mass, which explained 87% of the variance (*p* < 0.001).

**Table 3:** Summary of hierarchical multivariable regression for muscular strength and lower-limb muscular power

	Predictor	β	*P*-value	*R* ^2^
Leg press, 10 RM	Model	0.901	< 0.001	0.813
	Body mass	0.901	< 0.001	
Bench press, 10 RM	Model	0.909	< 0.001	0.826
	Body mass	0.600	< 0.001	
	Chronological age	0.347	0.007	
CMJa jump height	Model	0.718	< 0.001	0.515
	LLV	0.718	< 0.001	
Handgrip, dom	Model	0.935	< 0.001	0.874
	Chronological age	0.303	0.022	
	FFM	0.655	< 0.001	

CMJa = countermovement jump with arm swing, LLV = Lean leg volume, FFM = Fat-free mass. Handgrip dom = handgrip strength dominant arm

### Relationship between handgrip strength and total muscle strength

**Fig. 1:**
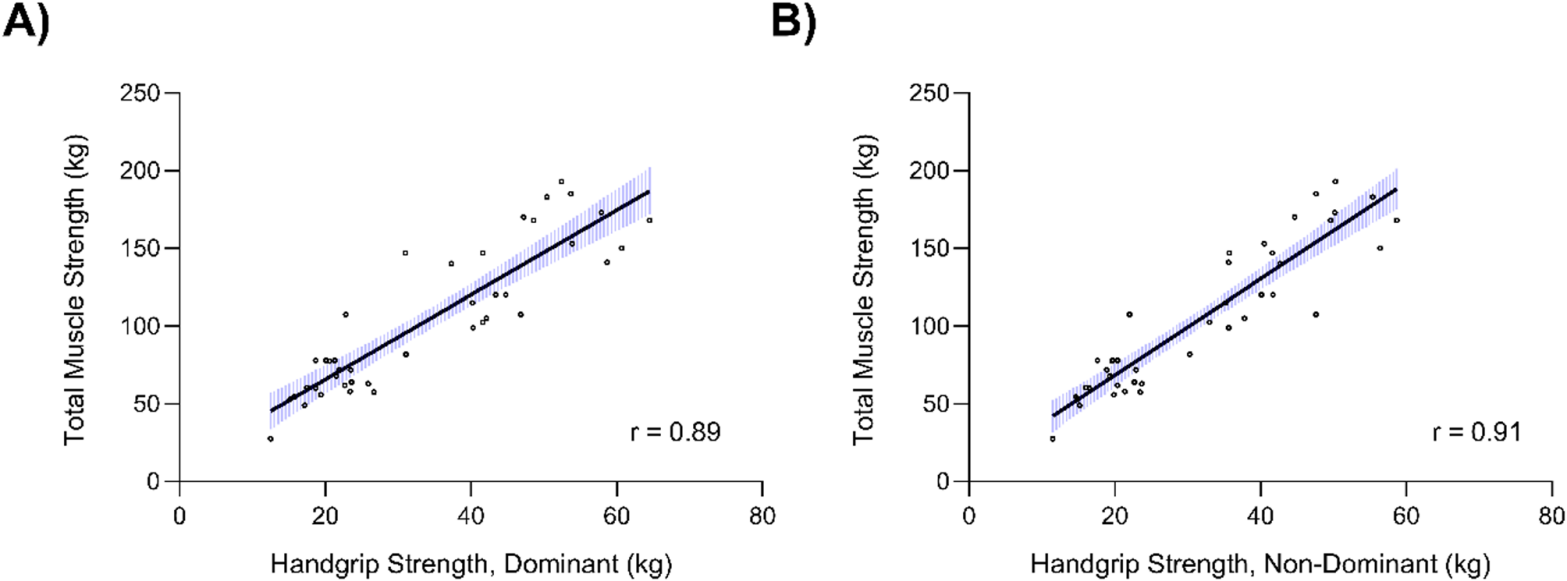
The relationship between total muscle strength (sum of leg press and bench-press strength) and (**A**) handgrip strength in the dominant arm and (**B**) handgrip strength in the non-dominant arm in trained male children (95% CI)

There was a strong correlation between total muscle strength and handgrip strength in the dominant (*r* = 0.89, *p* < 0.001). and non-dominant arms (*r* = 0.91, *p* < 0.001). The correlation between handgrip strength and total muscle strength is shown in Fig. 1. Similar correlation coefficients between handgrip strength and total muscle strength were observed in the prepubertal (handgrip non-dominant, *r* = 0.76, *p* < 0.001; handgrip dominant arm, *r* = 0.72, *p* < 0.001) and pubertal group (handgrip non-dominant, *r* = 0.75, *p* < 0.001; handgrip dominant, *r* = 0.61, *p* < 0.001).

## Discussion

This study shows that anthropometrical factors, rather than age and maturation variables, are the strongest independent factors in the model associated with muscular strength in trained male children. The factors associated with muscular strength vary significantly depending on the specific exercise test used, which is an important consideration for coaches and practitioners when assessing and comparing the strength levels of young children. While body mass appeared to be the most influential factor in leg press and bench press, FFM was the most significant determinant of handgrip muscle strength. On the other hand, the CMJa jump height was mostly related to lean leg volume. Lastly, a strong relationship was found between handgrip strength, and total muscle strength assessed by loaded upper- and lower-body resistance training exercises, suggesting that researchers and practitioners may use a simple handgrip muscle strength test to monitor trained male children’s muscle strength.

### Factors associated with muscle strength

In all multivariable regression analyses we conducted, anthropometric factors were the most important factor explaining muscle strength and power. Other studies have similarly reported strong relationships between height and body mass with isokinetic leg strength [13, 35]. However, it is important to note that most previous studies examining factors influencing strength development in youth have focused on isokinetic muscle strength [36], and our data on free-weight and machine exercise provide novel insights into the literature. Additionally, there was a variation between the selected tests. Body mass explained 81% of the variance in the 10RM leg press test (Table 3). Similarly, body mass showed the strongest association with the 10RM bench press, and the addition of chronological age explained 83% of the variation of strength (Table 3). On the other hand, FFM showed the strongest association with handgrip strength, and together with chronological age, it explained 87% of the variance in strength (Table 3). Our results are in line with previous research that shows that anthropometrical factors, including fat-free mass and body mass, are major determinants of muscle strength in children and adolescents [3, 13, 16, 36–38].

Our data align with previous studies, showing that muscle strength development appears to be highly related to muscle action and joint-specific; hence, transferring knowledge to other muscle activities may not be comparable [36]. Our data indicate that muscle strength in these age groups is primarily attributed to changes in body mass for leg press and bench press strength, whereas fat-free mass (FFM) is the primary determinant of handgrip strength. In contrast, lean leg volume had the strongest association with CMJa jump height performance, reflecting the importance of the primary muscles involved in jumping, as observed in other studies [19].

Furthermore, we found moderate- to strong associations between biological maturation and muscle strength (range: *r* = 0.84–0.89) and lower-limb muscle power (*r* = 0.64). However, when examined concurrently with other variables, biological maturation was no longer a significant independent variable explaining muscle strength. Supporting data from previous studies show that maturation does not exert an independent effect once height and body mass are added to the model to explain muscle strength in children and adolescents [39]. Similarly, a study using allometric scaling models in 14-16-year-old basketball players demonstrated that the exponents for body mass and thigh volume were greatly reduced by including maturation in the model, reinforcing the need to examine potential variables concurrently [13]. Hence, although there is a strong correlation between strength and maturation, a large portion of this association is probably attributed to variation in muscle growth (cross-sectional area) and anatomical growth (limb length) rather than maturation itself.

Muscle strength in male children increases with age, with a marked increase between 13 and 15 years old, when most children go through maturation before it plateau [40]. Some researchers have reported that age exerts an independent effect on strength development in children [3], while others have shown that age is no longer an independent factor of strength when examined concurrently with other anthropometric variables [39]. Our data only showed that age was an independent factor along with body mass for bench-press and handgrip strength, not other tests. Although age typically correlates highly to muscle strength, anatomical growth and maturation rates vary independently, and their effects on strength do not correlate simply with chronological age [3].

### Influence of maturation on muscle strength

Our findings showed significant increases in absolute muscular strength (leg press, bench press, and handgrip) and lower-limb power (CMJa) with advancing maturity status. However, when normalized using ratio- and allometric scaling, these differences between prepubertal and pubertal children were significantly reduced or eliminated. This suggests that the apparent influence of maturation on physical fitness outcomes is primarily a consequence of changes in body size and composition, particularly fat-free mass and lean leg volume, rather than maturation per se.

These results are consistent with previously published data, which clearly show that anthropometrical factors, such as height and body mass, are among the main determinants of muscle strength and power during childhood [41–43]. Additionally, our study also showed that ratio scaling to body mass generally overestimated the strength differences between maturation groups. These data further support previous studies showing that ratio scaling is ineffective for scaling body size and may produce misleading child-adolescent comparisons [44]. Previous research has suggested that coaches, educators, and clinicians should avoid using unscaled or ratio-scaled strength data to assess children’s strength levels instead of allometrically scaled data [41]. Allometrically scaled data enables children to be compared with strength norms while controlling body size. In contrast, with unscaled data, the smaller children would always seem weaker at any age [41].

The results are consistent with increasing literature showing that maturity-related differences in strength, power, and endurance often diminish when adjusted for more suitable anthropometric factors [13, 39, 45]. For example, longitudinal studies in 11-18-year-olds have shown that after allometric scaling (body mass and fat-free mass), maturity-related differences in mean power output and peak power output decrease substantially, highlighting the confounding role of size rather than maturity itself [46]. Physiological performance improvements can be attributed to the simultaneous development of musculoskeletal size (cross-sectional area, limb length), neuromuscular coordination, and training exposure that occurs during growth. These factors, rather than maturation alone, drive enhancements in performance. Therefore, assessing maturation without considering body size may lead to overestimating its role in strength development. These findings highlight the importance of examining anthropometric variables alongside maturation markers to more accurately interpret training outcomes and identify talent.

Lastly, accurate strength assessments with maturation may require multiple scaling approaches, as the choice of scaling variable significantly affects interpretation, particularly for tests involving lower-limb muscle power, such as the countermovement jump (CMJa). Overall, this has implications for both sports coaches who want to compare the strength levels of their young athletes or those involved in talent identification, as well as healthcare professionals examining pediatric populations and risk for disease or injury.

### Relationship between handgrip strength and total muscle strength

We observed a strong correlation between handgrip and total muscle strength in trained male children. To our knowledge, no previous study has examined the relationship between handgrip strength and total muscle strength, as assessed with loaded resistance training exercises, in an athletic youth population. Other studies have focused on healthy children and single-joint strength tests [27, 47]. Wind et al. [27] examined healthy children, adolescents, and young adults. They reported a strong correlation ranging from 0.73 to 0.89 between handgrip muscle strength and total muscle strength measured by summing up shoulder abductors, hip flexors and ankle dorsiflexion [27]. Another study reported significant correlation coefficients, *r* = 0.50, between grip strength and back strength and grip strength and quadriceps strength of *r* = 0.53 in healthy females [47]. Low handgrip muscle strength in youth has previously been shown to be associated with elevated metabolic risk factors [6]. Based on the existing literature, handgrip strength can be used as a simple method to predict total muscle strength from both a health and an athletic perspective. Predicting total muscle strength with a simple handgrip strength is useful for researchers and practitioners since it can be used as a quick scan to determine a child’s strength level for monitoring purposes for both health and performance. However, if the practitioner aims to individualize training loads for each child, more extensive testing batteries for different muscle groups, along with relevant anthropometrical tests, are needed.

### Limitations

Although identifying factors that influence muscle strength is important for coaches and practitioners who regularly conduct muscle fitness tests in children of varying body sizes, composition, and maturity, much additional work needs to be done. For example, we did not include robust measures of muscle cross-sectional area (CSA), which can be measured non-invasively with ultrasound techniques known to influence muscle strength [36]. Additionally, research is needed to investigate the relationship between neuromuscular function and muscle strength in children [36]. Our study also included a relatively homogeneous group of trained male children; extrapolation to untrained youth or females should be further investigated. The study employs an observational design, which inherently shares the same limitations as any other observational design, namely that it does not allow for a cause-and-effect relationship to be established. Lastly, no a priori power analysis was conducted since the study used pre-collected data that, in best practice, should be used.

### Practical application

Our results have several important implications for youth athletes’ sports performance, health monitoring, and talent development. Firstly, the findings underscore that muscular strength in trained male children and adolescents is more accurately predicted by anthropometric variables, particularly body mass, and fat-free mass, rather than by age or biological maturity alone. This challenges the common practice in youth sports where grouping by chronological age is used as well as by more recent “biobanding” (e.g. grouping based on biological age) [48].

For coaches and strength and conditioning professionals, this means that physical assessments should incorporate body size and composition measures to make fair comparisons between athletes and individualized training. For example, two athletes of the same maturity level may show different strength levels due to differences in lean mass or limb mechanics, which may have implications for determining training loads, recovery, and injury risk. Additionally, the reduced independent effect of biological maturation after scaling suggests that biobanding strategies in youth sports should be complemented with body size and composition assessments. Practitioners should avoid relying solely on unscaled or ratio-scaled data, which can overemphasize maturity-related differences and misclassify an athlete’s true strength capacity. Furthermore, handgrip strength demonstrated a strong correlation with total muscle strength, as assessed through loaded upper- and lower-body resistance exercises. This may provide a simple, cost-effective screening tool for youth populations. However, it should not replace comprehensive testing where precision is required for performance development of rehabilitation planning.

### Future perspectives

Longitudinal studies are needed to clarify how performance improvements align with growth and maturation over time. Further research should also explore the specificity of different strength assessments and investigate how neuromuscular factors interact with anthropometry across developmental stages.

## Conclusion

Anthropometric variables are the strongest factors associated with muscular strength in trained male children and adolescents, surpassing the influence of age and biological maturation. These findings support the use of body-size-adjusted assessments in both research and applied sports settings to assess and enhance muscular development. Handgrip strength appears to be a useful and efficient proxy for overall muscular fitness in this group.

## Supplementary Information

Below is the link to the electronic supplementary material.

Supplementary Material 1

## Data Availability

The datasets generated during and analyzed during the current study are available from the corresponding author upon reasonable request.

## Abbreviations

RM: Repetition maximum
CMJa: Countermovement jump with arms swing
FFM: Fat-free mass
LLV: Lean leg volume
CV: Coefficient of variation
VIF: Variance inflation factor
