# Supplementary material for: Factors associated with muscle strength in 10–16-year-old trained male children and adolescents
Source: BMC Sports Sci Med Rehabil. 2025 Aug 15;17:238. doi: 10.1186/s13102-025-01272-6 (PMC12355766; doi:10.1186/s13102-025-01272-6)
Supplement: Supplementary file 1 — Supplementary Material 1 [file 13102_2025_1272_MOESM1_ESM.docx]

**Supplementary Digital Content (SDC)**

**Table S1.** Univariable regression of muscular strength

| **Leg press, 10 RM** | | | | |  | **Bench press, 10 RM** | | | |
| --- | --- | --- | --- | --- | --- | --- | --- | --- | --- |
|  | N | β | r^2^ | *p* |  | N | β | r^2^ | *p* |
| Body mass | 41 | 0.90 | 0.81 | < 0.001 |  | 41 | 0.88 | 0.78 | < 0.001 |
| Height | 41 | 0.81 | 0.65 | < 0.001 |  | 41 | 0.79 | 0.63 | < 0.001 |
| FFM | 41 | 0.88 | 0.76 | < 0.001 |  | 41 | 0.91 | 0.82 | < 0.001 |
| LLV | 41 | 0.88 | 0.78 | < 0.001 |  | 41 | 0.87 | 0.76 | < 0.001 |
| Years of training | 41 | 0.70 | 0.49 | < 0.001 |  | 41 | 0.67 | 0.45 | < 0.001 |
| Tanner | 41 | 0.84 | 0.70 | < 0.001 |  | 41 | 0.89 | 0.79 | < 0.001 |
| Age | 41 | 0.79 | 0.62 | < 0.001 |  | 41 | 0.84 | 0.71 | < 0.001 |
| Leg length | 41 | 0.68 | 0.47 | < 0.001 |  | 41 | 0.74 | 0.55 | < 0.001 |
| Body Fat % | 41 | 0.28 | 0.08 | 0.079 |  | 41 | 0.44 | 0.19 | 0.004 |
| **Handgrip, dominant arm** | | | | |  | **CMJ** | | | |
| Body mass | 41 | 0.90 | 0.80 | < 0.001 |  | 41 | 0.61 | 0.37 | < 0.001 |
| Height | 41 | 0.88 | 0.77 | < 0.001 |  | 41 | 0.66 | 0.44 | < 0.001 |
| FFM | 41 | 0.93 | 0.86 | < 0.001 |  | 41 | 0.66 | 0.44 | < 0.001 |
| LLV | 41 | 0.93 | 0.86 | < 0.001 |  | 41 | 0.72 | 0.52 | < 0.001 |
| Years of training | 41 | 0.62 | 0.39 | < 0.001 |  | 41 | 0.58 | 0.34 | < 0.001 |
| Tanner | 41 | 0.88 | 0.78 | < 0.001 |  | 41 | 0.64 | 0.41 | < 0.001 |
| Age | 41 | 0.89 | 0.76 | < 0.001 |  | 41 | 0.69 | 0.48 | < 0.001 |
| Leg length | 41 | 0.77 | 0.60 | < 0.001 |  | 41 | 0.60 | 0.36 | < 0.001 |
| Body fat % | 41 | 0.48 | 0.22 | 0.002 |  | 41 | 0.41 | 0.17 | 0.008 |

FFM = fat free mass, LLV = lean leg volume, CMJ = countermovement jump
